# Biochemical failure after radical prostatectomy with PSA ≤ 1 ng/mL: prediction of PSMA-positive metastatic disease

**DOI:** 10.1007/s12149-026-02153-9

**Published:** 2026-01-15

**Authors:** Giulia Santo, Helena Rosarno, Antonino Restuccia, Giuseppe Lucio Cascini, Francesco Grillone, Francesco Cicone

**Affiliations:** 1 Department of Experimental and Clinical Medicine, “Magna Graecia” University of Catanzaro, Campus “Salvatore Venuta”, Viale Europa, Catanzaro, 88100 Italy; 2 Nuclear Medicine Unit, “Mater Domini” Hospital, “Renato Dulbecco” University Hospital, Catanzaro, Italy; 3 Department of Medicine and Heath Sciences “Vincenzo Tiberio”, University of Molise, Campobasso, Italy; 4 Medical Oncology Unit, “Pugliese-Ciaccio” Hospital, “Renato Dulbecco” University Hospital, Catanzaro, Italy

**Keywords:** Prostate cancer, Biochemical recurrence, PSMA, PET, Metastatic disease

## Abstract

**Objectives:**

The identification of metastatic disease in patients with biochemical failure of prostate cancer (PCa) after radical prostatectomy (RP) determines subsequent treatment management. Our objectives were (i) to assess the prevalence of metastatic PCa in patients with biochemical failure following RP and PSA levels ≤ 1 ng/mL, as detected by ^18^F-PSMA-1007 PET/CT (PSMA-PET), and (ii) to identify predictors of metastatic disease.

**Methods:**

Fifty-five patients with biochemical recurrence (BCR, *n* = 47) or persistent disease (*n* = 8) following RP as their primary and only prior treatment were retrospectively included if presenting with PSA levels ≤ 1 ng/mL. Patients who received any other anticancer treatment were excluded. PSMA-PET findings were categorized as either local recurrence (i.e., prostatic fossa and seminal vesicles) or metastases. Predictors of PSMA-PET positivity were assessed with univariate and multivariate regression analyses both in the whole sample and in two subgroups defined according to PSA levels (Group A = PSA < 0.5 ng/mL; Group B = PSA between 0.5 and 1 ng/mL).

**Results:**

Median PSA at the time of PET/CT was 0.37 ng/mL (range: 0.13–1.0). PSMA-PET was positive in 22/55 (40%) patients, 14/55 (25%) patients had metastatic disease. Overall, 31 PSMA-positive lesions were identified: 12/31 (39%) and 19/31 (61%) were local recurrences and metastases, respectively. In the whole cohort, ISUP grade > 3 (*p* = 0.003), pN1 status after surgery (*p* = 0.011), time to BCR ≤ 26 months (*p* = 0.03), and persistent disease (*p* = 0.003) were significantly associated with a higher rate of PSMA-positive metastases. At multivariate analysis, ISUP grade > 3 (*p* = 0.016) was the only independent predictor of metastases. Metastatic disease was detected in 8/38 (21%) patients in Group A and in 6/17 (35%) patients in Group B, respectively. In Group A, pN1 (*p* = 0.043) and persistent disease (*p* = 0.040) were significant predictors of metastases. In Group B, ISUP grade > 3 was the only predictor (*p* = 0.028).

**Conclusions:**

ISUP grade > 3, time to BCR ≤ 26 months, pN1 status, and persistent disease after surgery indicated a higher likelihood of PSMA-positive metastatic disease in a homogeneous cohort of patients with biochemical failure and low PSA values. pN1 status and persistent disease were significant predictors of metastatic disease also in patients with PSA levels < 0.5 ng/mL.

**Supplementary Information:**

The online version contains supplementary material available at 10.1007/s12149-026-02153-9.

## Introduction

Prostate cancer (PCa) is the most common malignancy and the third leading cause of cancer-related death among men in Europe [1]. Localized PCa is primarily treated with radical prostatectomy (RP), external beam radiotherapy (EBRT), or brachytherapy [2]. However, 20–50% of patients experience biochemical failure, including persistent disease or biochemical recurrence (BCR) within 10 years after definitive treatment [3–5].

In patients who underwent RP, BCR has been traditionally defined as a rise in serum prostate-specific antigen (PSA) levels above 0.2 ng/mL, confirmed by two consecutive measurements [6]. The most recent National Comprehensive Cancer Network (NCCN) guidelines consider a PSA increase above 0.1 ng/mL after RP as indicative of BCR [7]. Differently, BCR after radical EBRT is defined by the “Phoenix criteria” as any PSA increase 2 ng/mL higher than the PSA nadir value, regardless of the serum concentration of the nadir [6]. According to the European Association of Urology (EAU), persistent disease is defined as detectable PSA ≥ 0.1 ng/mL within 4–8 weeks after RP [2].

In patients with biochemical failure who have not undergone adjuvant radiotherapy, the treatment of choice is currently salvage RT (sRT) [6]. For sRT, the delineation of target volume is often performed without evidence of macroscopic recurrence, and it is usually based on regions with the highest likelihood of recurrence in different risk groups. sRT covers the prostatic bed in very low/low-risk group, extends to the seminal vesicles in intermediate risk group, and to the pelvic lymphatic drainage in high/very-high risk group [8]. It is well acknowledged that the efficacy of sRT is higher when applied at lower PSA levels [9]. The detection of sites of recurrence, particularly outside the standard fields of irradiation, could inform clinical decision-making, guide therapeutic choices, potentially leading to escalation or de-escalation of subsequent treatments. Escalation can be accomplished by increasing radiation volume, dose, or by adding a dose boost at the site of recurrence, whereas de-escalation could imply the omission of adjuvant systemic therapy [10–14]. The efficacy of emerging metastasis-directed therapies on patient outcomes is currently being evaluated by ongoing randomized trials [15, 16].

Prostate-specific membrane antigen positron emission tomography/computed tomography (PSMA-PET) has become a well-established imaging modality for detecting recurrence in PCa patients after radical treatment. PSMA-PET showed superior diagnostic performance for the identification of distant sites of disease compared to conventional magnetic resonance imaging (MRI) [17, 18] as well as to other radiopharmaceuticals such as ^18^F-fluoromethylcholine [19, 20] and ^18^F-fluciclovine [21]. The high sensitivity of PSMA-PET, especially for patients with PSA levels lower that 1 ng/mL, has been particularly emphasized [19–22], as the largest clinical impact on therapy planning is expected in this patient population. In fact, patients with limited disease burden may still be subject to optimized locoregional therapies, whereas sRT alone is generally considered suboptimal, if not futile, in patients with high likelihood of systemic disease, i.e. those with PSA levels above 1 ng/mL [23].

Most of the available studies on the diagnostic performances of PSMA-PET in patients with biochemical failure have included heterogeneous cohorts with a wide range of PSA levels at the time of PET and various previous treatments, including RP, adjuvant/salvage EBRT, and systemic therapies [24–33]. This heterogeneity limits the assessment of the impact of PSMA-PET in patients who may benefit most from localized therapies.

The PSMA-PET detection rate of metastatic disease at low PSA levels (< 1 ng/ml) has been reported by several authors, however most often with similar limitations [12, 34–41]. Furthermore, factors associated with the occurrence of PSMA-positive metastatic disease in this setting have been rarely investigated [34–36]. Aims of the present study were (i) to assess the prevalence of metastatic PCa in patients with biochemical failure following RP and PSA levels ≤ 1 ng/mL, as detected by ^18^F-PSMA-1007 PET/CT and (ii) to identify predictive factors associated with the detection of PSMA-positive metastatic disease.

## Materials and methods

All consecutive patients with PCa referred for ^18^F-PSMA-1007 PET/CT at the Nuclear Medicine Unit of the “Mater Domini” University Hospital of Catanzaro (IT) between July 2021 and April 2025 were reviewed. To be included in the final analysis, patients were required to have BCR or persistent disease following RP as their primary and only prior treatment, with PSA levels between 0.1 and 1.0 ng/mL. Both BCR and persistent disease were defined according to the latest EAU guidelines [2, 6]. Patients who had undergone any other anticancer treatment, including adjuvant or salvage radiotherapy and/or androgen deprivation therapy (ADT) as well as all patients with PSA > 1.0 ng/mL were excluded from the analysis. The following clinical data were retrospectively extracted from medical records: age, date of RP, total PSA levels at diagnosis (initial PSA, iPSA) and at the time of BCR (PSA – PET), time to BCR, presence of persistent disease after RP, as well as histopathological findings after RP (Gleason Score, ISUP grade, pTNM classification, and resection margin status). PSA doubling time (PSAdt) and PSA velocity (PSAve) were calculated using the tool available on the Memorial Sloan Kettering Cancer Center website [42]. Follow-up data after PSMA-PET were retrieved from a prospectively maintained database available at the institutional clinical oncology department. The study was performed in accordance with the ethical standards of the 1964 Declaration of Helsinki and later amendments. Written informed consent to use data for research purposes was obtained from all patients. The retrospective evaluation of patients’ imaging and clinical data was approved by the institutional ethical board (Ethical Committee Regione Calabria, prot. 276/2025).

### ^18^F-PSMA-1007 PET/CT imaging

Whole-body PET/CT images were acquired 90 min after the intravenous injection of 3.6–4.4 MBq/kg of ^18^F-PSMA-1007 (RADELUMIN^®^), covering the region from the skull base to the proximal thighs. Although the excretion of ^18^F-PSMA-1007 is predominantly hepatobiliary (up to 95%) with only about 5% renal excretion, a delayed post-void pelvic acquisition was performed only in cases of uncertain findings in the prostatic bed, where physiological radioactive urine could interfere with image interpretation, as previously suggested [43, 44]. All acquisitions were performed using a GE-Healthcare Discovery ST 8-slice camera, operating in 2D mode. Image reconstruction was performed using the ordered subset expectation maximization (OSEM) algorithm with 2 iterations and 30 subsets, applying a 5 mm Gaussian smoothing filter post-reconstruction. The PET reconstruction parameters were as follows: field of view (FOV): 60 × 60 × 29.1 cm³. Matrix size: 128 × 128 × 89. Voxel size: 4.7 × 4.7 × 3.27 mm³. The co-registered low-dose CT scan (60 mA, 120 kV) was reconstructed with FOV: 50 × 50 × 29.1 cm³, Matrix size: 512 × 512 × 89 Voxel size: 0.98 × 0.98 × 3.27 mm³. All relevant corrections were applied, including normalization, dead-time correction, activity decay correction, random coincidence correction, attenuation correction, and scatter correction.

### ^18^F-PSMA-1007 PET/CT image analysis

All ^18^F-PSMA-1007 PET/CT scans were retrospectively reviewed by two independent readers (G.S. and H.R.) blinded to clinical data. Images suspicious of disease involvement were identified based on the interpretation criteria proposed by Werner et al. [45] and in accordance with the current EANM guidelines [46]. Increased radiotracer uptake was considered suspicious for malignancy if meeting the following criteria: (a) the uptake was greater than the surrounding background and could not be attributed to physiological distribution and (b) the lesion was located in anatomical regions compatible with prostate cancer dissemination.

PET-detected disease was categorized as either local recurrence (i.e., prostatic fossa and seminal vesicles) or distant metastatic disease (i.e., pelvic lymph nodes, extrapelvic lymph nodes, and bone).

### Statistical analysis

Statistical analyses were performed using IBM SPSS Statistics version 30.0 (IBM Corp., Armonk, NY, USA), and GraphPad Prism version 10.5 (GraphPad Software, Boston, MA, USA). Categorical and continuous variables were analyzed using descriptive statistics. Multiple univariate regression analyses were performed to identify predictors of PSMA-PET positivity. Variance inflation factor (VIF) was used to exclude multicollinearity. The main focus of the paper was to identify factors associated with the presence of metastatic disease. However, predictors of overall PSMA-PET positivity in the whole cohort were also analyzed and corresponding results were reported as supplementary material. Significant predictors at univariate analysis were incorporated into a multivariable logistic regression model. Multiple univariate analyses were additionally conducted to identify potential predictors of metastatic disease in two different patient subgroups stratified according to the total PSA values: Group A included patient with PSA levels in the range 0.1–0.49 ng/mL, while Group B included patient with PSA levels between 0.50 and 1.00 ng/mL.

All continuous variables were dichotomized according to their median values. Categorical variables were dichotomized as follows: ISUP grade (≤ 3 vs. >3), pathological T stage (pT2 vs. pT3), resection margin status (R0 vs. R1), and persistent disease (yes/no). Pathological N stage included patients with pN0, pN1 and pNx. Given that most patients with pNx (i.e., 95%) fell into the low-risk ISUP grade group (Supplementary Table 1), pNx patients were assimilated to pN0 and compared with patients with pN1 stage. Probability values of less than 0.05 were considered statistically significant.

## Results

A total of 174 patients were screened for inclusion. Seventy-five patients presenting with PSA values > 1.0 ng/mL were excluded from the analysis. Thirty-five patients who were not at first relapse (i.e., metastatic patients, castration resistant), as well as 9 patients who had undergone radical or salvage radiotherapy were also excluded. A total of 55 patients met the inclusion criteria. The main characteristics of the patients included in the study are summarized in Table 1. A total of 8 patients were found to have persistent disease after RP (Table 2). On post-prostatectomy histological examination: 34 patients (61.8%) had a pT3 and 19 (34.5%) had pT2 stage, 7 patients (12.7%) had lymph node involvement (pN1), and 25 patients (45.5%) had positive surgical margins. Following current guidelines [6, 7], PSMA PET was the first-line imaging modality at recurrence in most patients, owing to its higher sensitivity and specificity compared with conventional morphological imaging. Nine out of 55 patients (16%) were referred for PSMA-PET after negative pelvic MRI (*n* = 8) or CT (*n* = 1).

**Table 1:** Patient characteristics

Variable	Population	N° of PSMA-PET positive patients
Age (years)	68 (49–76)	/
iPSA (ng/mL)	7.38 (3.38–85.46)	/
PSA – PET (ng/mL)	0.37 (0.13–1.0)	/
PSAdt (ng/mL/month)	8.62 (1–28.35)	/
PSAve (ng/mL/year)	0.37 (0–3.42)	/
Time from BCR to PSMA PET (months)	26 (1–195)	/
*ISUP Grade (%)*		
ISUP 1	5 (9.1%)	1
ISUP 2	21 (38.2%)	8
ISUP 3	18 (32.7%)	5
ISUP 4	8 (14.5%)	5
ISUP 5	3 (5.5%)	3
*Pathologic T stage (%)*		
pT2	19 (34.5%)	7
pT3	34 (61.8%)	15
Not available	2 (3.6%)	0
*Pathologic N stage (%)*		
pN0	27 (49.1%)	11
pN1	7 (12.7%)	5
pNx	19 (34.5%)	6
Not available	2 (3.6%)	0
*Surgical margins status (%)*		
R0	27 (49.1%)	11
R1	25 (45.5%)	10
Not available	3 (5.5%)	1
*Persistent disease after RP (%)*		
No	47 (85.5%)	16
Yes	8 (14.5%)	6

BCR = biochemical recurrence; iPSA = initial PSA; ISUP = International Society of Urological Pathology; PET = positron emission tomography; PSA = prostate-specific antigen; PSAdt = PSA doubling time; PSMA = prostate-specific membrane antigen; PSAve = PSA velocity; RP = radical prostatectomy. For continuous variables median values (range) are reported. For categorical variables, number of patients (percent) are reported

**Table 2:** Characteristics of patients with persistent disease after radical prostatectomy

Patient	Age RP	ISUP grade	Time from RP (weeks)	PSA value (ng/mL)	PET result	Metastatic disease	Site of disease on PSMA PET
1	61	4	5	0.258	+	yes	Pararectal L
2	63	3	5	0.32	–		
3	69	2	6	0.34	+	yes	Prostatic fossa + pararectal R
4	69	3	8	0.43	–		
5	63	3	8	0.476	+	yes	Obturator R
6	62	5	7	0.98	+	yes	Obturator L
7	70	5	6	1.0	+	yes	Prostatic fossa + V rib L + vertebrae L3 + hip L
8	68	4	4	1.0	+	yes	Prostatic fossa + pararectal R + internal iliac R

ISUP = International Society of Urological Pathology; L = left side; PET = positron emission tomography; PSA = prostate-specific antigen; PSMA = prostate-specific membrane antigen; R = right side; RP = radical prostatectomy

### Prevalence of positive PSMA PET in the overall cohort and in different subgroups

**Fig. 1:**
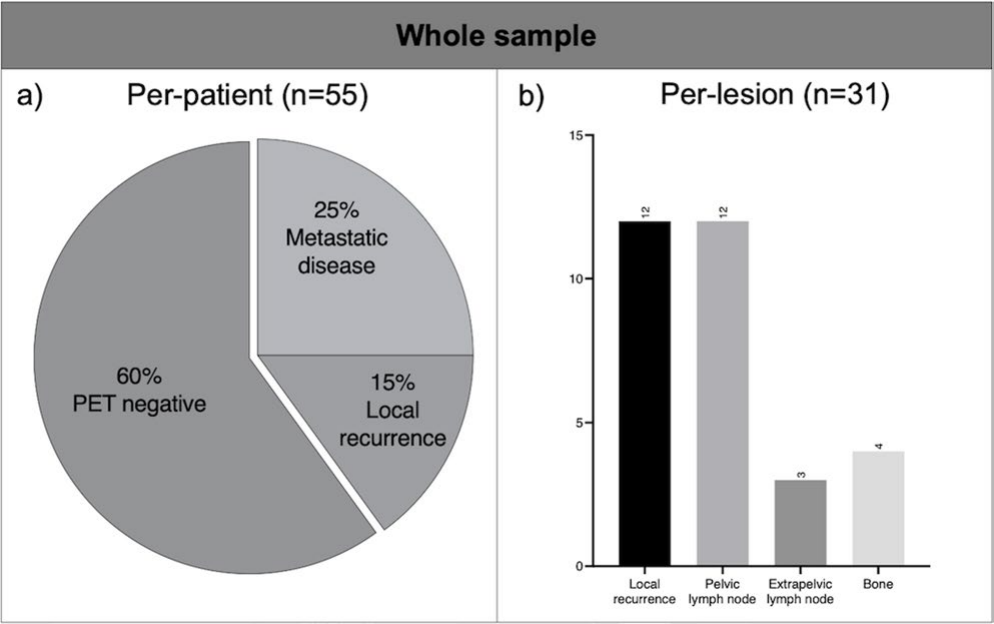
PSMA positivity in the entire cohort (**a**) and regional distribution of PSMA-positive lesions (**b**)

PSMA-PET was positive in 22/55 (40%) patients, detecting metastatic disease in 14/55 (25%) patients. Overall, PSMA-PET was able to identify a total of 31 PSMA positive lesions: 12/31 (39%) were local recurrences (*n* = 11 prostatic bed, *n* = 1 seminal vesicles), and 19/31 (61%) lesions were considered metastatic disease (*n* = 12 regional lymph nodes; *n* = 3 extra-regional lymph nodes; *n* = 4 bone lesions) (Fig. 1). Eight patients showed local recurrence only, 7 patients showed abnormal uptake only in pelvic lymph nodes, 2 patients had PSMA-positive disease in the prostatic fossa and in the pelvic lymph nodes. Moreover, 2 patients showed pathological uptake in the fossa, pelvic and extrapelvic lymph nodes, and in 1 patient one extrapelvic lymph node was involved. Bone uptake was seen in 2 patients with (*n* = 1) or without (*n* = 1) a positive lesion in the prostatic fossa, respectively.

**Fig. 2:**
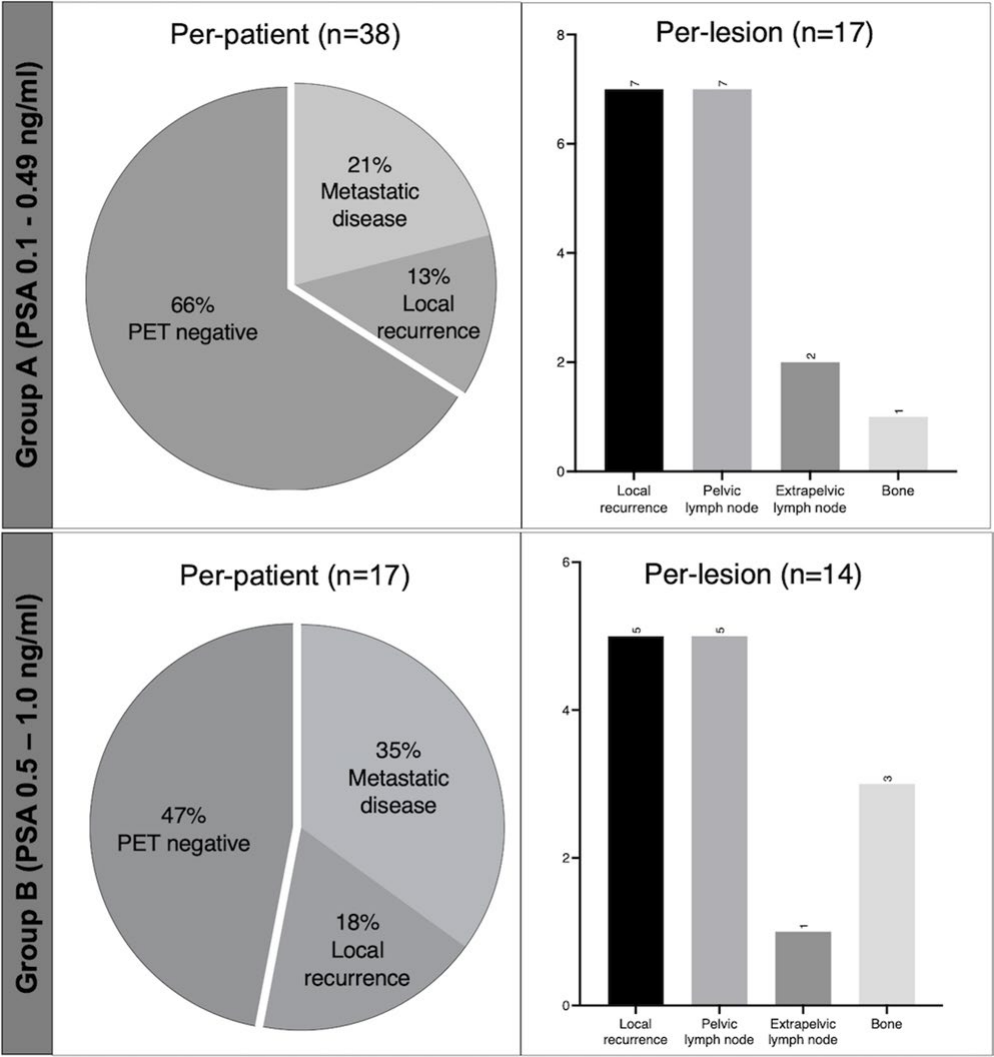
Distribution of PSMA-positive lesions in the subgroups (Group A and Group B), defined according to the total PSA values

A total of 38 patients were referred for PSMA-PET with PSA value below 0.5 ng/mL (Group A). Thirteen of these patients (34%) had a positive PSMA-PET: 5 patients showed local recurrence only, while 8 had metastatic disease. A total of 17 lesions were detected in this subgroup. Group B included 17 patients with PSA between 0.5 and 1.0 ng/mL. Nine (53%) of these patients showed a positive PSMA-PET; 6 of these patients had metastatic lesions. Figure 2 shows the distribution of PSMA-positive lesions in the subgroups (Group A and Group B), defined according to the total PSA values.

**Fig. 3:**
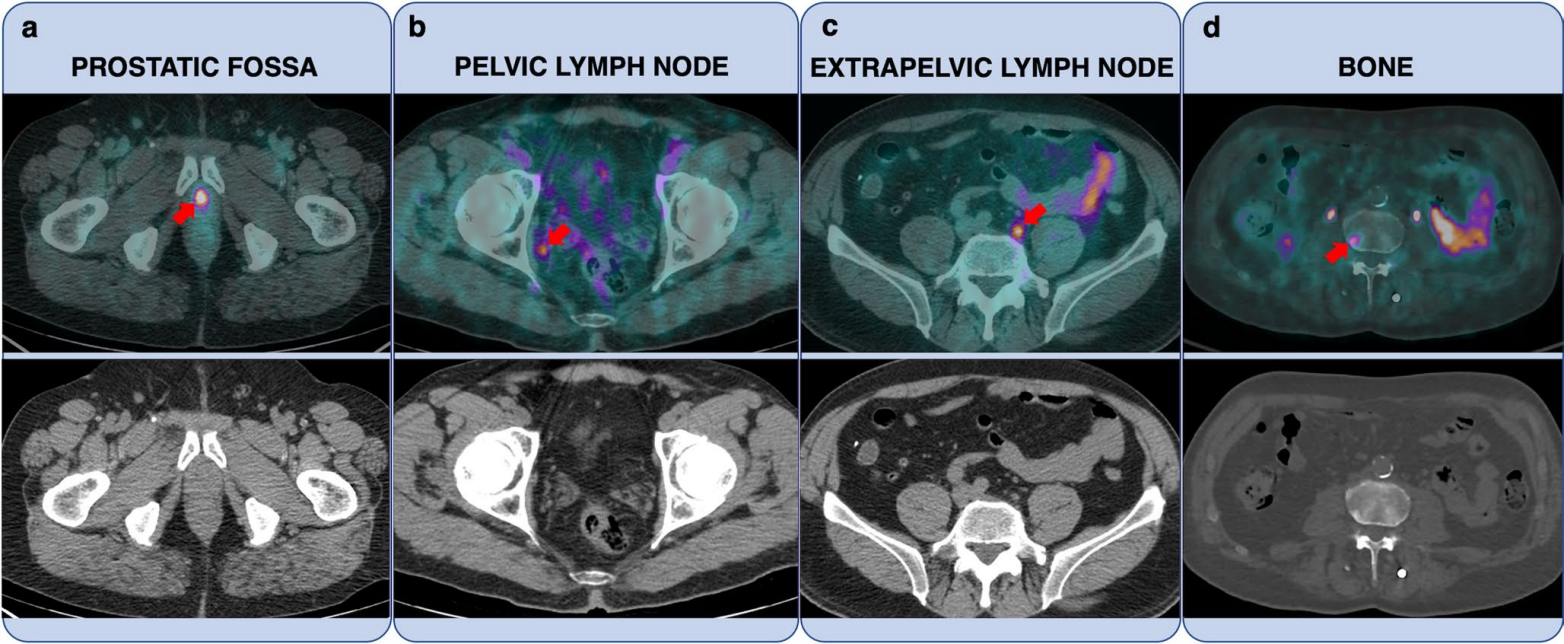
Examples of PSMA-PET/CT positive disease. (**a**) A 63-year-old patient with biochemical recurrence (BCR) (PSA = 0.37 ng/mL) of PCa occurred 9 months after radical prostatectomy (RP). PSMA PET identified a focal uptake in the prostatic fossa (red arrow). (**b**) A 73-year-old patient with BCR (PSA = 0.23 ng/mL) 24 months after RP. PSMA PET identified a pelvic lymph node in the right obturator region (red arrow). (**c**) A 67-year-old patient with BCR (PSA = 0.91 ng/mL) 8 months following RP. PSMA PET revealed metastatic disease in the left common iliac region (red arrow). (**d**) A 71-year-old patient with BCR (PSA = 0.31 ng/mL) 26 months after RP. PSMA PET showed bone disease in the right hemibody of D5 (red arrow)

No significant difference was found between Groups A and B regarding overall PSMA-PET positivity (34% vs. 53% in Group A and B, respectively, *p* = 0.19, χ² test) or detection of distant metastases (21% vs. 35%, *p* = 0.26, χ² test). Overall PSMA-PET positivity and detection of distant metastases did not differ significantly in both subgroups even when patients with persistent disease were excluded from the analysis (overall PET positivity: 30% vs. 43%, *p* = 0.60 χ² test; detection of distant metastases: 15% vs. 21%, *p* = 0.40, χ² test in Group A and B, respectively). Figure 3 describes some case examples extracted from our cohort.

### Predictive factors for detection of PSMA-positive metastatic disease

#### Overall cohort

Results of the univariate and multivariate regression investigating factors associated with overall PSMA-PET positivity are shown in Supplementary Table 2.

The univariate analysis revealed that ISUP grade > 3 (*p* = 0.003), pathological nodal disease (pN1) after surgery (*p* = 0.011), time to BCR ≤ 26 months (*p* = 0.003), and persistent disease after surgery (*p* = 0.003), were significantly associated with a higher rate of PSMA-positive metastatic disease (Table 3). The multivariate model identified ISUP grade > 3 (*p* = 0.016) as the only independent predictors of metastatic disease on PSMA-PET. By excluding the *n* = 8 patients with persistent disease from the analysis, ISUP grade > 3 (Odd Ratio (OR): 7.246, 95%CI 1.047–50.144, *p* = 0.045) and pN1 (OR: 18.566, 95%CI, 1.232–279.728, *p* = 0.035) remained significant predictors of metastatic localization on PSMA-PET (Supplementary Table 3).

**Table 3:** Univariate and multivariate analysis for predictors of PSMA-positive metastatic disease in the whole sample

Parameters	Metastatic disease			
	Univariate		Multivariate	
	Odds Ratio (95%CI)	*p*	Odds Ratio (95%CI)	*p*
iPSA (≤ 7.38 vs. >7.38)	0.360 (0.094–1.374)	0.135		
PSA – PET (≤ 0.37 vs. >0.37)	1.544 (0.454–5.248)	0.487		
ISUP grade (≤ 3 vs. >3)	9.250 (2.126–40.242)	**0.003**	8.436 (1.495–47.613)	**0.016**
pT stage (pT2 vs. pT3)	1.562 (0.415–5.890)	0.510		
pN status (pN0/pNx vs. pN1)	10.278 (1.709–61.826)	**0.011**	7.513 (0.837–67.443)	0.072
Resection margin (R0 vs. R1)	0.750 (0.218–2.578)	0.648		
Time to BCR (≤ 26 vs. >26 months)	0.193 (0.047–0.799)	**0.023**	0.486 (0.087–2.726)	0.412
PSAdt (≤ 8.63 vs. >8.63 months)	0.400 (0.102–1.572)	0.189		
PSAve (≤ 0.37 vs. >0.37 ng/mL/year)	1.565 (0.418–5.864)	0.507		
Persistent disease (no/yes)	14.625 (2.486–86.025)	**0.003**	3.965 (0.432–36.391)	0.223

BCR = biochemical recurrence; iPSA = initial PSA; ISUP = International Society of Urological Pathology; PET = positron emission tomography; PSA = prostate-specific antigen; PSAdt = PSA doubling time; PSMA = prostate-specific membrane antigen; PSAve = PSA velocity. Bold font indicates statistical significance.

### Group A: PSA value 0.1–0.49 ng/mL

Metastatic disease was detected in 8 out of 38 (21%) patients with PSA < 0.5 ng/mL. In this subgroup, pathological nodal disease (pN1) after surgery (*p* = 0.043), and persistent disease after surgery (*p* = 0.040) were confirmed as predictors of PET positivity for metastatic disease. Table 4 shows results of the univariate regression analysis in Group A.

**Table 4:** Predictors of PSMA positive metastatic disease in the group A

Parameters	Univariate	
	Odds Ratio (95%CI)	*p*
iPSA (≤ 7.38 vs. >7.38)	0.325 (0.054–1.958)	0.220
ISUP grade (≤ 3 vs. >3)	5.400 (0.838–34.800)	0.076
pT stage (pT2 vs. pT3)	1.354 (0.271–6.758)	0.712
pN status (pN0/pNx vs. pN1)	8.10 (1.066–61.536)	**0.043**
Resection margin status (R0 vs. R1)	1.071 (0.224–5.128)	0.931
Time to BCR (≤ 26 vs. >26 months)	0.143 (0.016–1.308)	0.085
PSAdt (≤ 8.63 vs. >8.63 months)	0.557 (0.110–2.810)	0.479
PSAve (≤ 0.45 vs. >0.45 ng/mL/year)	1.700 (0.346–8.344)	0.513
Persistent disease (no/yes)	8.400 (1.107–63.735)	**0.040**

Notes: BCR = biochemical recurrence; iPSA = initial PSA; ISUP = International Society of Urological Pathology; PET = positron emission tomography; PSA = prostate-specific antigen; PSAdt = PSA doubling time; PSMA = prostate-specific membrane antigen; PSAve = PSA velocity. Bold font indicates statistical significance.

### Group B: PSA value 0.5–1.0 ng/mL

Metastatic disease was detected in 6 out of 17 (35%) patients with PSA levels between 0.5 and 1.0 ng/mL. In this subgroup, ISUP grade > 3 was the only factor significantly associated with the detection of metastases (*p* = 0.028). Table 5 shows results of the univariate regression analysis in Group B.

**Table 5:** Predictors of PSMA positive metastatic disease in the group B

Parameters	Univariate	
	Odds Ratio (95%CI)	*p*
iPSA (≤ 7.38 vs. >7.38)	0.400 (0.047–3.424)	0.403
ISUP grade (≤ 3 vs. >3)	20.00 (1.391–287.600)	**0.028**
pT stage (pT2 vs. pT3)	1.250 (0.089–17.653)	0.869
pN status (pN0/pNx vs. pN1)	ND	ND
Resection margin status (R0 vs. R1)	0.400 (0.047–3.424)	0.403
Time to BCR (≤ 26 vs. >26 months)	0.111 (0.011–1.094)	0.060
PSAdt (≤ 8.63 vs. >8.63 months)	0.167 (0.012–2.368)	0.186
PSAve (≤ 0.37 vs. >0.37 ng/mL/year)	0.857 (0.055–13.479)	0.913
Persistent disease (no/yes)	ND	ND

BCR = biochemical recurrence; iPSA = initial PSA; ISUP = International Society of Urological Pathology; PET = positron emission tomography; PSA = prostate-specific antigen; PSAdt = PSA doubling time; PSMA = prostate-specific membrane antigen; PSAve = PSA velocity. ND = not determined because of unbalanced groups. Bold font indicates statistical significance.

### Patient follow-up

All patients (*n* = 8) showing PSMA-positive disease limited to the prostatic fossa underwent sRT without the addition of ADT. Of the 9 patients with pelvic lymph node involvement, 3/9 underwent sRT alone, 3/9 underwent sRT plus ADT, 2/9 received sRT to the pelvis plus stereotactic body radiotherapy (SBRT) to the PSMA-detected lymph nodes and ADT. In one patient a “watch and wait” strategy was adopted.

All three patients with extrapelvic lymph-node involvement and the two patients with bone metastases received a combined approach of sRT plus ADT. In one case, SBRT to the involved lymph-nodes was added.

## Discussion

PSMA-PET is recognized as a highly accurate imaging technique for restaging PCa patients with biochemical failure after radical treatment, which led to its inclusion in major international oncological guidelines [6]. It has been demonstrated that the early detection of recurrent disease, particularly outside the prostatic fossa, can influence subsequent treatment planning by identifying metastases in areas not covered by standard clinical target volumes (CTVs). In a retrospective evaluation of 140 patients with BCR, Emmet et al. showed that most treatment failures occurred in those patients with PSMA positive metastatic disease not covered by CTVs [47]. In a cohort of 270 patients with BCR and PSA levels lower than 1 ng/mL, Calais et al. demonstrated that 19% of patients had at least one ^68^Ga-PSMA-11 positive lesion not covered by the consensus CTVs. In nearly 40% of cases, these metastatic lesions were found within the pelvis and most often occurred in perirectal lymph nodes [12].

The primary objective of our study was to evaluate the prevalence of PSMA-positive metastatic disease in a homogeneous cohort of patients with biochemical failure after RP and PSA levels below 1 ng/mL. In our cohort, the median PSA level was 0.37 ng/mL, and the prevalence of PSMA-PET positive patients was 40%. The percentage of patients with metastatic disease was 25%, which falls within the range (i.e., 18–45%) reported by others [12, 34, 35]. Potential sources of heterogeneity between studies include the concurrent use of ADT at the time of PSMA-PET imaging [38, 39, 48], which may exert a time-dependent effect on PSMA expression [49], or differences in image interpretation criteria or in the PSMA ligands used for PET/CT [24, 25, 28–30].

To our knowledge, only two prior studies have used similar inclusion criteria, both using ^68^Ga-PSMA-11 for PET/CT imaging [40, 41]. Van Leeuwen et al. studied 70 patients with BCR and PSA ≤ 1 ng/mL [40], while Miksch et al. included 116 patients with BCR and PSA ≤ 0.6 ng/mL after RP [41]. Overall positivity rates were 54.3% and 50% in [40] and in [41], respectively.

In our study 61% of PSMA-positive lesions were metastases, while 39% were local recurrences, which align well with the findings of Van Leeuwen et al. (i.e., 57.2% distant metastases and 42.8% local recurrences) [40]. Metastatic lesions were mostly located in pelvic lymph nodes, whereas extrapelvic lymph nodes and bone metastases were identified only in a small proportion of patients. In contrast, Miksch et al. found a slightly higher prevalence of local recurrences compared to metastatic lesions [41]. Since the prostatic fossa is the most common site of recurrence at first biochemical relapse, small discrepancies may be partially explained by urinary tracer excretion, which can obscure lesions at the vesicourethral anastomosis, which is the most frequent site of recurrence, as well as at the bladder neck, or in the bladder lumen [50]. As a matter of fact, it has been shown that MRI outperforms PSMA-PET for detecting local recurrence (sensitivity 91% vs. 64%, specificity 95% vs. 74%, accuracy 92% vs. 78%) [51], and that proximity to the bladder is strongly associated with false-negative PET results [52].

The second objective of our study was to identify potential predictors of PSMA-positive metastatic disease in the entire cohort and in subgroups stratified according to PSA levels. The choice of a PSA cutoff value of 0.5 ng/mL to identify two different subgroups is not arbitrary. According to the major clinical guidelines, this is the threshold above which the initiation of salvage therapy is recommended [53].

A higher rate of metastatic disease was associated with the presence of persistent disease after RP both in the overall cohort and among patients with PSA ≤ 0.5 ng/mL. The inclusion of patients with persistent disease in previous cohorts has been heterogeneously reported [29, 32, 54–56]. In our population, 6/8 (i.e., 75%) patients with persistent disease showed a PSMA-positive metastatic lesion. In a previous study including 150 patients with biochemical persistence after RP, 67% of patients showed a positive PSMA-PET. Of those, 59% showed PSMA-positive disease outside the prostatic fossa [56]. Similar results were reported by others [55]. Taken together, these findings appear clinically relevant, as it is known that 5–20% of patients exhibit persistent PSA levels following RP [57], and most of these patients may indeed have distant metastases. This is the reason why patients with persistent disease following RP and PSA levels as low as 0.2 ng/mL should be systematically assessed with PSMA-PET [53]. It remains to be determined whether a pre-surgical staging with PSMA-PET might have an impact on the survival outcomes of these patients, given that it is likely that the presence of metastatic disease preceded RP [56].

Additional significant predictors of the presence of PSMA-positive metastatic disease in our cohort were a time to BCR ≤ 26 months and an ISUP grade greater than 3, the latter being the only variable that remained significant at multivariate analysis. Similar results were obtained by the group of Bernardino et al., who studied a heterogeneous cohort of 137 patients with BCR and PSA levels ≤ 1 ng/mL, identifying a Gleason Group of 4–5 as an independent predictor of metastases on ^18^F-DCFPyL PSMA PET/CT (OR 6.43; 95% CI 1.90–21.82; *p* = 0.003) [34]. Of note, it has been shown by several groups that shorter BCR and higher Gleason Score, among others, are associated with an increased rate of PSMA-PET positivity, but the association with metastatic disease has so far rarely been investigated [33, 39, 58, 59].

Lastly, the presence of pN1 stage was also a significant predictor of metastatic disease. This was confirmed both in the overall cohort and in patients with PSA ≤ 0.5 ng/mL. A significantly higher detection rate of metastatic disease was found in pN1 patients compared with both pN0 patients (71% vs. 30%; *p* = 0.043, χ² test) and pNx patients (71% vs. 5%; *p* < 0.001, χ² test). For the logistic regression model, we decided to group pNx patients together with pN0 patients. The choice was based on the expected low likelihood of metastatic disease in patients with pNx in our cohort, who mostly (i.e., 95%) fell into the low-risk ISUP grade group (See Supplementary Table 1). This hypothesis was confirmed by the higher detection rate of PSMA-positive metastatic disease found in pN0 compared with pNx patients (30% vs. 5%, *p* = 0.040, χ² test).

There is a lack of consensus on the optimal management of patients with pN1 [60], and several strategies are currently accepted based on patient-specific risk assessment. For patients with only 1 or 2 involved lymph nodes and undetectable postoperative PSA, both active monitoring and immediate adjuvant RT combined with systemic hormonal therapy are considered acceptable options [61, 62]. It should be reminded that, because of our inclusion criteria, our population included only patients with lower-risk disease that were not initially referred to adjuvant radiotherapy. It is noteworthy that 5/7 (71.4%) patients with pN1 disease showed PSMA-positive metastases, suggesting that an early diagnostic and/or therapeutic intervention might be beneficial even in these patients.

Our study has several limitations that should be acknowledged. Most importantly, the relatively small sample size limited the statistical power of our analysis, and other predictors of metastatic disease in the general population could have been missed in our sample. For example, in our study, the PSA doubling time, a known marker or disease aggressiveness, did not reach statistical significance, either in the overall population or within subgroups. Additionally, the number of patients classified as pN1 (i.e., *n* = 7) was unbalanced between subgroups. Furthermore, histological confirmation of findings was not available, however, in clinical practice, biopsy is not always feasible or appropriate, and previously published studies on PSMA-PET have compared imaging findings with histological gold standard in a negligible number of patients [39, 40, 59].

## Conclusions

The prevalence of PSMA PET-detected metastatic disease in patients with BCR of PCa after RP and PSA levels ≤ 1 ng/mL was 25%. ISUP grade greater than 3 and time to BCR ≤ 26 months were associated with the detection of metastatic disease in the overall cohort. Persistent disease after RP and pN1 status were significant predictors of metastatic disease both in the overall cohort and in the subgroup of patients with PSA ≤ 0.5 ng/mL.

This suggests that, in presence of these negative prognostic factors, an early imaging assessment with PSMA-PET may be considered even for PSA levels which do not currently mandate initiation of salvage therapy, as it might have a significant impact on subsequent management. However, larger prospective studies with homogeneous patient cohorts are needed to confirm our preliminary findings.

## Supplementary Information

Below is the link to the electronic supplementary material.

Supplementary material 1
