# Correction: Biochemical failure after radical prostatectomy with PSA ≤ 1 ng/mL: prediction of PSMA-positive metastatic disease

**DOI:** 10.1007/s12149-026-02238-5

**Published:** 2026-05-25

**Authors:** Giulia Santo, Helena Rosarno, Antonino Restuccia, Giuseppe Lucio Cascini, Francesco Grillone, Francesco Cicone

**Affiliations:** 1 Department of Experimental and Clinical Medicine, “Magna Graecia” University of Catanzaro, Campus “Salvatore Venuta”, Viale Europa, Catanzaro, 88100 Italy; 2 Nuclear Medicine Unit, “Mater Domini” Hospital, “Renato Dulbecco” University Hospital, Catanzaro, Italy; 3 Department of Medicine and Heath Sciences “Vincenzo Tiberio”, University of Molise, Campobasso, Italy; 4 Medical Oncology Unit, “Pugliese-Ciaccio” Hospital, “Renato Dulbecco” University Hospital, Catanzaro, Italy

**Correction to: Annals of Nuclear Medicine (2026) 40:521–531**

10.1007/s12149-026-02153-9

In this article, the caption for Fig. 3(d) should be replaced with: ‘A 70-year-old patient with persistent disease 4 weeks after RP (PSA = 1.0 ng/mL). PSMA PET showed bone disease in the left fifth rib, left hip, and right hemibody of L3 (red arrow).’

