# Supplementary material for: Biochemical failure after radical prostatectomy with PSA ≤ 1 ng/mL: prediction of PSMA-positive metastatic disease
Source: Ann Nucl Med. 2026 Jan 15;40(5):521–31. doi: 10.1007/s12149-026-02153-9 (PMC13124769; doi:10.1007/s12149-026-02153-9)
Supplement: Supplementary file 1 — Supplementary material 1 [file 12149_2026_2153_MOESM1_ESM.docx]

*Annals of Nuclear Medicine*

**Biochemical Failure After Radical Prostatectomy with PSA ≤ 1 ng/mL: Prediction of PSMA-positive Metastatic Disease**

Giulia Santo^1^, Helena Rosarno^2^, Antonino Restuccia^2^, Giuseppe Lucio Cascini^3^, Francesco Grillone^4^, Francesco Cicone^1,2^

^1^Department of Experimental and Clinical Medicine, "Magna Graecia" University, Catanzaro, Italy

^2^Nuclear Medicine Unit, “Mater Domini" Hospital, “Renato Dulbecco” University Hospital, Catanzaro, Italy

^3^Department of Medicine and Heath Sciences “Vincenzo Tiberio”, University of Molise, Campobasso, Italy

^4^Medical Oncology Unit, “Pugliese-Ciaccio” Hospital, “Renato Dulbecco” University Hospital, Catanzaro, Italy

**Corresponding Author:**

Prof. Francesco Cicone, MD, Phd. ORCID ID 0000-0003-4664-1965

Department of Experimental and Clinical Medicine, “Magna Graecia” University, Campus "Salvatore Venuta", Viale Europa - 88100 Catanzaro, Italy. Email: cicone@unicz.it. Tel : 0039(0)9613694155

**Supplementary Table 1.** Baseline characteristics of Nx subpopulation

| **Variable** | **Nx subpopulation** |
| --- | --- |
| **Age (years)** | 68 (49 – 74) |
| **iPSA (ng/mL)** | 6.50 (3.38 – 11.50) |
| **PSA – PET (ng/mL)** | 0.35 (0.15 – 1.0) |
| **PSAdt (ng/mL/month)** | 13.36 (1 – 122.7) |
| **PSAve (ng/mL/year)** | 0.097 (0 – 1.685) |
| **Time from BCR to PSMA PET (months)** | 43 (10 – 195) |
| **ISUP Grade (%)**  **ISUP 1**  **ISUP 2**  **ISUP 3**  **ISUP 4** | 3 (16%)  11 (58%)  4 (21%)  1 (5%) |
| **Pathologic T stage (%)**  **pT2**  **pT3** | 11 (58%)  8 (42%) |
| **Surgical margins status (%)**  **R0**  **R1**  **Not available** | 7 (37%)  11 (58%)  1 (5%) |
| **Persistent disease after RP (%)**  **No**  **Yes** | 19 (100%)  0 (0) |

Notes: BCR= biochemical recurrence; iPSA= initial PSA; ISUP= International Society of Urological Pathology; PET=positron emission tomography; PSA= prostate-specific antigen; PSAdt = PSA doubling time; PSMA= prostate-specific membrane antigen; PSAve = PSA velocity; RP=radical prostatectomy. For continuous variables median values (range) are reported. For categorical variables, number of patients (percent) are reported.

**Supplementary Table 2.** Univariate and multivariate analysis for predictors of PSMA positivity in the entire cohort, including both local recurrences and metastatic disease.

| **Parameters** | PSMA PET positivity (positive vs. negative) | | | |
| --- | --- | --- | --- | --- |
|  | Univariate | | Multivariate | |
|  | OR (95%CI) | *p* | OR (95%CI) | *p* |
| **iPSA (≤7.38 vs. >7.38)** | 0.549 (0.176 – 1.717) | 0.303 |  |  |
| **PSA – PET (≤0.37 vs. >0.37)** | 1.440 (0.487 – 4.255) | 0.509 |  |  |
| **ISUP grade (≤3 vs. >3)** | 5.714 (1.313 – 24.871) | **0.020** | 4.413 (0.955 – 20.387) | 0.057 |
| **pT stage (pT2 vs. pT3)** | 1.353 (0.428 – 4.283) | 0.607 |  |  |
| **pN status (pN0/pNx vs. pN1)** | 4.265 (0.744 – 24.436) | 0.103 |  |  |
| **Resection margin (R0 vs. R1)** | 0.970 (0.320 – 2.939) | 0.957 |  |  |
| **Time to BCR (**≤**26 vs. >26 months)** | 0.421 (0.139 – 1.277) | 0.127 |  |  |
| **PSAdt (≤8.63 vs. >8.63 months)** | 0.709 (0.224 – 2.244) | 0.559 |  |  |
| **PSAve (≤0.37 vs. >0.37 ng/ml/year)** | 1.410 (0.446 – 4.464) | 0.559 |  |  |
| **Persistent disease (no/yes)** | 5.812 (1.051 – 32.145) | **0.044** | 4.059 (0.669 – 24.637) | 0.128 |

Notes: BCR=biochemical recurrence; iPSA =initial PSA; ISUP= International Society of Urological Pathology; PET=positron emission tomography; PSA= prostate-specific antigen; PSAdt= PSA doubling time; PSMA=prostate-specific membrane antigen; PSAve= PSA velocity. Bold font indicates statistical significance.

**Supplementary Table 3.** Univariate and multivariate analysis for predictors of PSMA-positive metastatic disease in the cohort of patients with BCR (n=47), after the exclusion of n=8 patients with persistent disease.

| **Parameters** | Metastatic disease | | | |
| --- | --- | --- | --- | --- |
|  | Univariate | | Multivariate | |
|  | Odds Ratio (95%CI) | *p* | Odds Ratio (95%CI) | *p* |
| **iPSA (≤7.38 vs. >7.38)** | 0.358 (0.061 – 2.092) | 0.245 |  |  |
| **PSA – PET (≤0.37 vs. >0.37)** | 1.167 (0.255 - 5.346) | 0.843 |  |  |
| **ISUP grade (≤3 vs. >3)** | 5.250 (0.898 - 30.700) | 0.066 | 7.426 (1.047 - 50.144) | **0.045** |
| **pT stage (pT2 vs. pT3)** | 1.136 (0.235 – 5.487) | 0.874 |  |  |
| **pN status (pN0/pNx vs. pN1)** | 12.000 (0.936 - 153.885) | 0.056 | 18.566 (1.232 -279.728) | **0.035** |
| **Resection margin (R0 vs. R1)** | 1.000 (0.216 - 4.628) | 1.000 |  |  |
| **Time to BCR (**≤**26 vs. >26 months)** | 0.375 (0.078 - 1.803) | 0.221 | 0.507 (0.087 - 2.961) | 0.450 |
| **PSAdt (≤8.63 vs. >8.63 months)** | 0.300 (0.051 – 1.763) | 0.183 |  |  |
| **PSAve (≤0.37 vs. >0.37 ng/mL/year)** | 1.583 (0.308 – 8.146) | 0.582 |  |  |

Notes: BCR=biochemical recurrence; iPSA =initial PSA; ISUP= International Society of Urological Pathology; PET=positron emission tomography; PSA= prostate-specific antigen; PSAdt= PSA doubling time; PSMA=prostate-specific membrane antigen; PSAve= PSA velocity. Bold font indicates statistical significance.
